# Cytogenetic and molecular aberrations and worse outcome for male patients in systemic mastocytosis

**DOI:** 10.7150/thno.51872

**Published:** 2021-01-01

**Authors:** Hanneke C. Kluin-Nelemans, Mohamad Jawhar, Andreas Reiter, Bjorn van Anrooij, Jason Gotlib, Karin Hartmann, Anja Illerhaus, Hanneke N.G. Oude Elberink, Aleksandra Gorska, Marek Niedoszytko, Magdalena Lange, Luigi Scaffidi, Roberta Zanotti, Patrizia Bonadonna, Cecelia Perkins, Chiara Elena, Luca Malcovati, Khalid Shoumariyeh, Nikolas von Bubnoff, Sabine Müller, Massimo Triggiani, Roberta Parente, Juliana Schwaab, Michael Kundi, Anna Belloni Fortina, Francesca Caroppo, Knut Brockow, Alexander Zink, David Fuchs, Irena Angelova-Fischer, Akif Selim Yavuz, Michael Doubek, Mattias Mattsson, Hans Hagglund, Jens Panse, Anne Simonowski, Vito Sabato, Tanja Schug, Madlen Jentzsch, Christine Breynaert, Judit Várkonyi, Vanessa Kennedy, Olivier Hermine, Julien Rossignol, Michel Arock, Peter Valent, Wolfgang R. Sperr

**Affiliations:** 1 Department of Haematology University Medical Center Groningen, University of Groningen, Groningen, The Netherlands.; 2 III. Medizinische Klinik, Universitätsmedizin Mannheim, Universität Heidelberg, Mannheim, Germany.; 3 Internal Medicine, section Allergology, University Medical Center Groningen, University of Groningen, Groningen, The Netherlands.; 4 Division of Hematology, Department of Medicine, Stanford University School of Medicine, Stanford, USA.; 5 Division of Allergy, Department of Dermatology, University of Basel, Basel, Switzerland (KH); *Department of Biomedicine, University of Basel, Basel, Switzerland.; 6 Department of Dermatology and Venerology, Uniklinik Köln, Köln, Germany.; 7 Department of Allergology, Medical University of Gdansk, Gdańsk, Poland.; 8 Department of Dermatology, Venereology and Allergology, Medical University of Gdansk, Gdańsk, Poland.; 9 Section of Hematology, Department of Medicine, Verona University Hospital, Verona, Italy.; 10 Allergy Unit, Verona University Hospital, Verona, Italy.; 11 Department of Molecular Medicine and Department of Hematology Oncology, University of Pavia and Fondazione IRCCS Policlinico San Matteo, Pavia, Italy.; 12 Department of Hematology, Oncology and Stem Cell Transplantation, Medical Center, Faculty of Medicine, University of Freiburg, Freiburg, Germany; German Cancer Consortium (DKTK) Partner Site Freiburg, Freiburg, Germany.; 13 Department of Hematology and Oncology, Medical Center, University of Schleswig-Holstein, Campus Lübeck, Lübeck, Germany.; 14 Department of Dermatology, Medical Center University of Freiburg, Faculty of Medicine, University of Freiburg, Germany; German Cancer Consortium (DKTK) Partner Site Freiburg, Freiburg, Germany.; 15 Division of Allergy and Clinical Immunology, University of Salerno, Salerno, Italy.; 16 Institute of Environmental Health, Medical University of Vienna, Vienna, Austria.; 17 Pediatric Dermatology Unit, Department of Medicine, University of Padova, Padova, Italy.; 18 Department of Dermatology and Allergy Biederstein, School of Medicine, Technical University of Munich, Munich, Germany.; 19 University Clinic for Hematology and Oncology, Kepler University Hospital, Johannes Kepler University, Linz, Austria.; 20 Department of Dermatology and Venereology, Allergy Centrr, Kepler University Hospital, Linz, Austria.; 21 Division of Hematology, Istanbul Medical School, University of Istanbul, Istanbul, Turkey.; 22 University Hospital and CEITEC Masaryk University, Brno, Czech Republic.; 23 Dept Immunol, Genetics and Pathology (MM) and Dept Hematol (MM, HH), Uppsala University Hospital, Uppsala University, Uppsala, Sweden.; 24 Department of Oncology, Hematology, Hemostaseology and Stem Cell Transplantation, University Hospital RWTH Aachen, Aachen, Germany.; 25 Faculty of Medicine and Health Sciences, Department of Immunology-Allergology-Rheumatology, University of Antwerp and Antwerp University Hospital, Antwerpen, Belgium.; 26 Department of Dermatology and Venereology, Medical University of Graz, Graz, Austria.; 27 Medical Clinic and Policlinic 1, Hematology and Cellular Therapy,Leipzig University Hospital,Germany.; 28 Department of Dermatology, University Hospitals Leuven, Leuven, Belgium.; 29 Department of Hematology, Semmelweis University, Budapest, Hungary.; 30 Division of Hematology/Oncology, Department of Medicine, University of California San Francisco, San Francisco, USA.; 31 French Reference Center for Mastocytosis (CEREMAST), Hôpital Necker, Assistance Publique Hôpitauxde Paris, Imagine Institute, University Paris Descartes, Paris, France.; 32 Department of Hematology, Gustave Roussy Cancer Center, Villejuif, France.; 33 Laboratory of Hematology, Pitié-Salpêtrière Hospital, Paris, France.; 34 Department of Internal Medicine I, Division of Hematology and Hemostaseology, Medical University of Vienna, Vienna, Austria.; 35 Ludwig Boltzmann Institute for Hematology and Oncology.

**Keywords:** Mastocytosis, sex difference, cytogenetics, molecular mutations, survival

## Abstract

In systemic mastocytosis (SM), the clinical features and survival vary greatly. Patient-related factors determining the outcome in SM are largely unknown.

**Methods:** We examined the impact of sex on the clinical features, progression-free survival (PFS), and overall survival (OS) in 3403 patients with mastocytosis collected in the registry of the European Competence Network on Mastocytosis (ECNM). The impact of cytogenetic and molecular genetic aberrations on sex differences was analyzed in a subset of patients.

**Results:** Of all patients enrolled, 55.3% were females. However, a male predominance was found in a subset of advanced SM (AdvSM) patients, namely SM with an associated hematologic neoplasm (SM-AHN, 70%; *p <* 0.001). Correspondingly, organomegaly (male: 23% *vs.* female: 13%, *p =* 0.007) was more, whereas skin involvement (male: 71% *vs.* female: 86%, *p =* 0.001) was less frequent in males. In all patients together, OS (*p <* 0.0001) was significantly inferior in males, and also within the WHO sub-categories indolent SM, aggressive SM (ASM) and SM-AHN. PFS was significantly (*p =* 0.0002) worse in males when all patients were grouped together; due to low numbers of events, this significance persisted only in the subcategory smoldering SM. Finally, prognostically relevant cytogenetic abnormalities (10% *vs.* 5%, *p =* 0.006) or molecular aberrations (*SRSF2*/*ASXL1*/*RUNX1* profile; 63% *vs.* 40%, *p =* 0.003) were more frequently present in males.

**Conclusions:** Male sex has a major impact on clinical features, disease progression, and survival in mastocytosis. Male patients have an inferior survival, which seems related to the fact that they more frequently develop a multi-mutated AdvSM associated with a high-risk molecular background.

## Introduction

Mastocytosis is a myeloid neoplasm presenting with an expansion and accumulation of neoplastic mast cells in one or more organ systems, such as the bone marrow (BM), skin, liver, spleen, and the gastrointestinal (GI) tract 1,2. The World Health Organization (WHO) classifies the disease into cutaneous mastocytosis (CM), indolent systemic mastocytosis (ISM), smoldering SM (SSM), SM with an associated hematologic neoplasm (SM-AHN), aggressive SM (ASM) and mast cell leukemia (MCL) 3-5. In daily routine, the term advanced SM (AdvSM) has been used to denote ASM, SM-AHN and MCL. Here, new developments including sensitive detection of the *KIT* D816V mutation and use of next-generation sequencing (NGS) panels have been of great help in understanding the multilineage basis and complex genetics of this category, especially SM-AHN 6.

Clinical characteristics and disease courses vary greatly from patient to patient, depending on the WHO subset, other disease-specific and patient-related factors, such as age or co-morbidities 7.The prognosis can change during follow-up, especially when the patient progresses to a more advanced type of disease 8,9.

A number of disease-specific and patient-related factors have been examined regarding their clinical and prognostic impact in SM 10-15. However, the impact of sex on the clinical course and outcome of mastocytosis remains largely unknown. We employed the dataset of the registry of the European Competence Network on Mastocytosis (ECNM) to examine the impact of sex on clinical features, progression and survival in 3403 patients with mastocytosis. In addition, we used data from a sub-cohort of a single ECNM center to investigate the impact of differences regarding the occurrence of cytogenetic and molecular aberrations in male and female patients with mastocytosis.

## Methods

### Diagnostic evaluations

Details about the ECNM registry have been published elsewhere 16. Although the registry is still recruiting, we used the data from a validated cohort updated in March 2019. Patients with mastocytosis were enrolled in 26 centers in Europe (12 countries) and one in the United States. The following parameters were documented: age, sex, height and weight, date of diagnosis, presence of major and minor diagnostic criteria according to the WHO classification 2008 and 2016 4, the final WHO category of mastocytosis, laboratory values at diagnosis and during follow-up, including blood counts and differentials, serum tryptase levels, the presence of hepatosplenomegaly and/or lymphadenopathy, the presence and severity of symptoms, including skin symptoms, flushing, osteoporosis, anaphylaxis, allergy and specific IgE, therapy, and responses including the use of symptomatic and cytoreductive drugs. The *KIT* D816V mutation was measured at local laboratories following the ECNM recommendations 17. *KIT* D816V variant allele frequency was only incidentally determined. If the *KIT* D816V mutation was absent, laboratories were strongly advised to search for other *KIT* mutations. In a subset of patients, molecular and cytogenetic data were collected. Cytogenetic analysis was performed at local laboratories, and consisted mainly of conventional methods. Molecular analysis using next generation deep amplicon sequencing (NGS) was performed as described 10. Adult patients with typical mast cell infiltrates in the skin, but without BM data and thus not fully classifiable according to WHO criteria, were included as cases with mastocytosis in the skin (MIS). Two patients with mast cell sarcoma collected in the registry were excluded in this study.

### Investigations during follow-up

Physicians were asked to update and check all included data yearly in AdvSM patients and every other year in cutaneous SM and ISM. The database of the ECNM registry, data storage and data distribution comply with the rules and regulations of data protection laws, with local ethics committee regulations of each participating center 16, and with the declaration of Helsinki.

In addition to data from the ECNM registry, we used data from a cohort of 190 AdvSM patients collected by a single ECNM center (Mannheim, Germany). In these cases, detailed molecular data were available. Out of these 190 patients, 90 were also included in the ECNM registry.

### Statistical analyses

The probability of overall survival (OS - time from diagnosis to death from any course) and progression-free survival (PFS) were determined by Kaplan and Meier estimates. PFS was defined as progression from one WHO category to another: from cutaneous to systemic; from ISM to SSM or AdvSM; within AdvSM to MCL; within SM-AHN categories from low grade myelodysplastic syndrome (MDS) to high grade MDS, and all transformations into secondary acute myeloid leukemia (AML). For PFS, patients classified as MIS (incomplete BM data) and MCL (no further progression possible) were excluded. Statistical significance of sex differences among distinct subtypes of the WHO classification was determined by a test of equal distribution by sex (50:50) calculating the exact binomial probability. A *p* value of *<* 0.05 was considered significant. Clinical symptoms captured in the registry were dichotomized: yes or no. GI symptoms were split into: stomach ulcer, cramping, and diarrhea. The impact of sex on differences in clinical characteristics was examined by determining odds ratio (OR) and 95% confidence intervals (CI) adjusted for WHO diagnostic categories in all patients.

### Data sharing statement

All data registered in the ECNM database are supervised by a registry consortium consisting of all participants who yearly meet, define the rules and regulations through which patient data are collected, projects are selected and conducted. The data are secured on the servers of the Austrian Control Bank. Individual participant data will not be shared by members outside the ECNM.

## Results

### Basic characteristics and sex distribution in the ECNM registry cohort

Data from 3403 patients were available of whom 44.7% were male (Table 1). Nineteen percent had CM, 50.8% ISM, 2.0% SSM, and 12.9% AdvSM. In 524 adult patients (15.4%), the pre-diagnostic term mastocytosis in the skin (MIS) was applied. Significant sex differences were observed in distinct subtypes of the WHO classification (Table 1). In the “MIS”, MPCM, and ISM groups, females were more frequent than males (MIS: 67% *vs.* 33%, *p <* 0.001; MPCM: 56% *vs*. 44%, *p =* 0.011; ISM: 56% *vs.* 44%; *p <* 0.001). In contrast, there was a statistically significant male predominance in the subgroup of patients with SM-AHN, whereas patients with ASM and MCL showed a trend towards male predominance (ASM, 52%, *p =* 0.77; SM-AHN, 70%, *p <* 0.001; MCL, 58%, *p =* 0.360).

The median age at diagnosis of male and female patients was 47.4 years (range, 0-91 years) and 44.4 years (range, 0-87 years), respectively, men being significantly older (*p* = 0.002). Median age at diagnosis per WHO subcategory and per sex is shown in Table 1. It appeared that in the AdvSM patients (grouped together) men were also significantly older (*p* < 0.0009), both in the ECNM registry and in the Mannheim cohort (Table 4). In Table 2, clinical features and symptoms related to mastocytosis are presented. Skin involvement (86% *vs*. 71%), GI symptoms (44% *vs*. 31%) and headache 16% *vs.* 8%) were significantly (all *p <* 0.0001) more often reported by female patients. In contrast, male patients more often had organomegaly (enlargement of spleen and/or liver and/or lymphadenopathy; 23% *vs.* 13%, *p <* 0.0001)). Anaphylaxis, allergy and osteoporosis did not show a sex preference (Table 2). In multivariate analysis, including the WHO subcategories, all these features and symptoms remained significant as far as sex difference was concerned (Table 2).

### Male mastocytosis patients have inferior outcome compared to female patients

**Figure 1:**
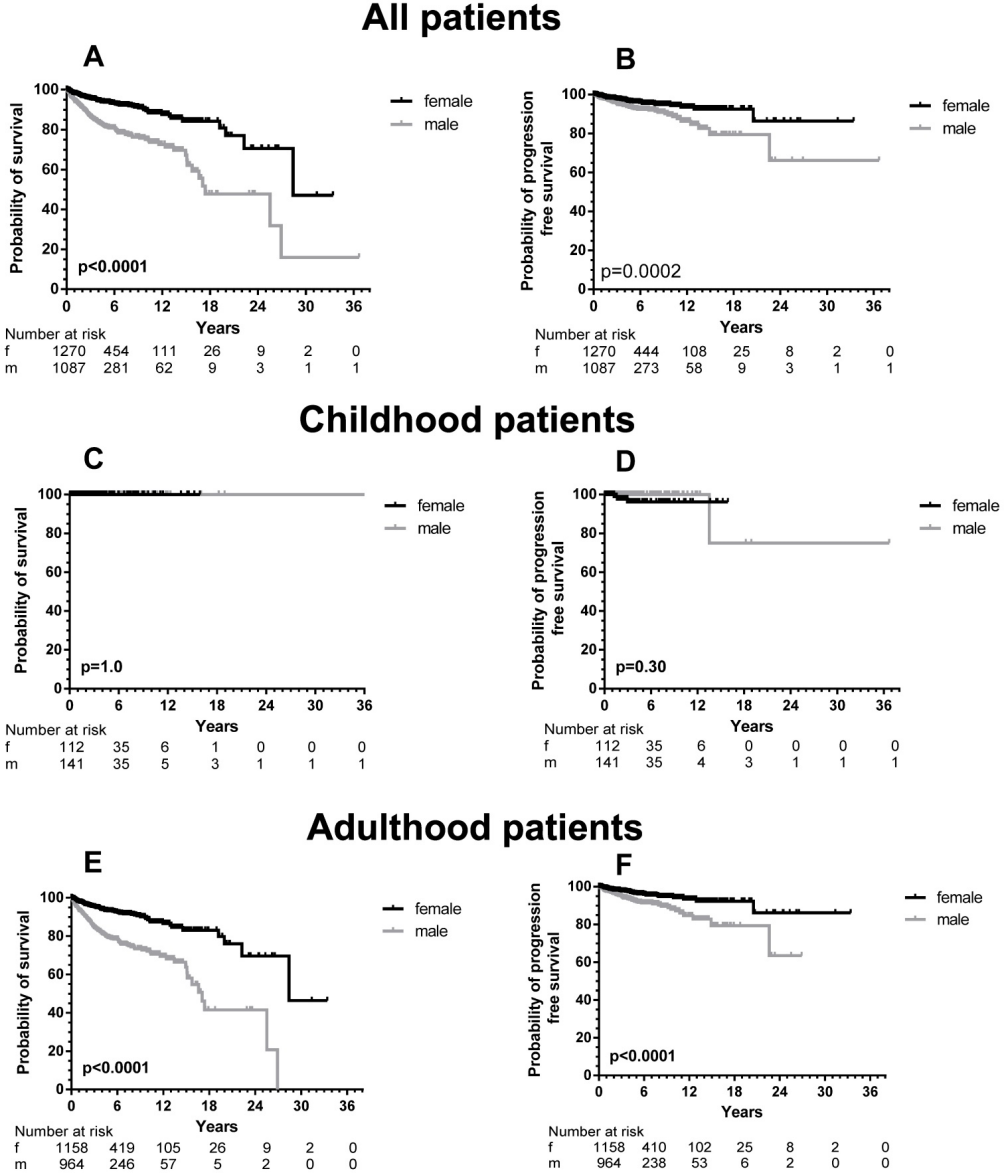
OS (left part, **A, C, and E**) and PFS (right part, **B, D, and F**) according to sex differences of all patients (adult and children), children only (C and D) and adults only (E and F) with mastocytosis specified according to male (grey line) and female (black line) patients. The numbers at risk are given at 6 years intervals.

**Figure 2:**
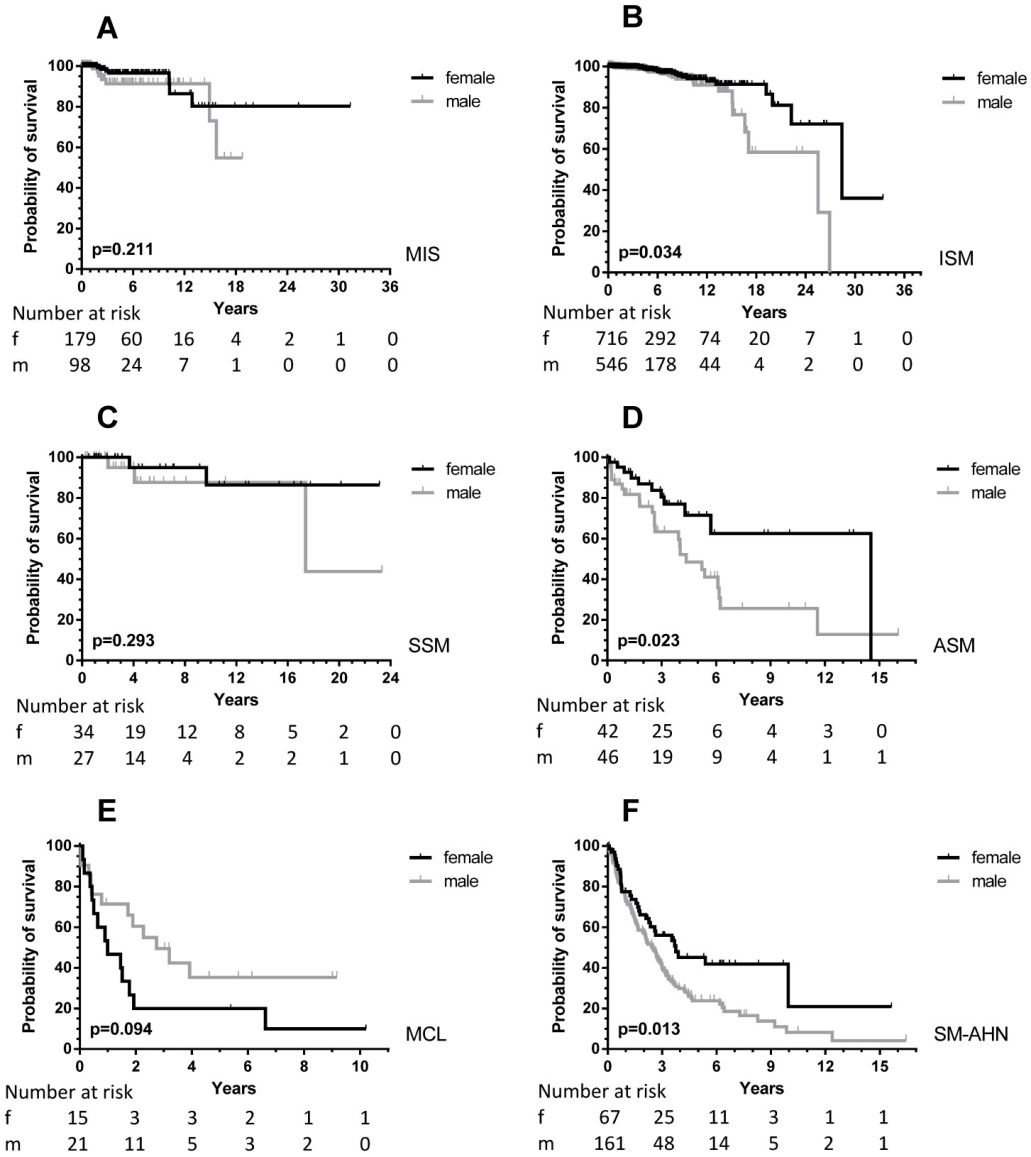
OS according to sex differences in the various WHO subcategories of all patients (adult and children) with SM. Grey lines: male patients; black lines: female patients. The numbers at risk are given at the time points outlined on the X-axis. **A:** MIS: adults with mastocytosis in the skin who did not undergo a BM analysis; **B:** ISM: indolent systemic mastocytosis; **C:** SSM: smoldering systemic mastocytosis; **D:** ASM: aggressive systemic mastocytosis; **E:** MCL: mast cell leukemia; **F:** SM-AHN: systemic mastocytosis with an associated hematological neoplasm. As there were no events in patients with cutaneous mastocytosis only (OS 100%), the curves are not shown.

Sufficient follow-up data, defined as any measure point at least 1 day from diagnosis were available in 2357 patients. There was a significant difference in OS between male and female patients (Figure 1, upper part) with a median OS difference of 11 years (17.4 *vs.* 28.4 years, *p <* 0.0001) in favor of female patients. Twelve-year OS for male patients was 72.5% (CI 67.3-77.0% and 87.5% for female patients (CI 83.5-90.5%). When we analyzed children and adults separately, the significance persisted as far as the adult patients are concerned (Figure 1, lower part). In children no differences were seen, because none of the children died during the follow-up. When the various WHO subcategories of mastocytosis were analyzed (Figure 2), the same pattern was seen in ISM, ASM and SM-AHN, but not in MCL. Here, male patients showed a trend towards a better survival (*p =* 0.095; Figure 2). The survival curves within the largest SM category, ISM, showed an interesting pattern, with overlapping curves during the first 10-15 years, after which more male patients died (Figure 2). Causes of death, however, were not different between both groups: 23 of 755 (3%) male patients with ISM died; causes of death were disease-related (n = 6, mostly progression or anaphylaxis-related deaths), other (n = 12), and unknown (n = 5). Twenty-two of 974 (2%) female ISM patients died; causes of death were disease-related (n = 5, mostly progression or anaphylaxis-related deaths), therapy-related (n = 1), other (n = 11), and unknown (n = 5). OS curves from adult and childhood patients with the WHO subcategory cutaneous mastocytosis (MPCM, DCM, mastocytoma) showed a 100% survival for both male and female patients.

### PFS differences between male and female patients

**Figure 3:**
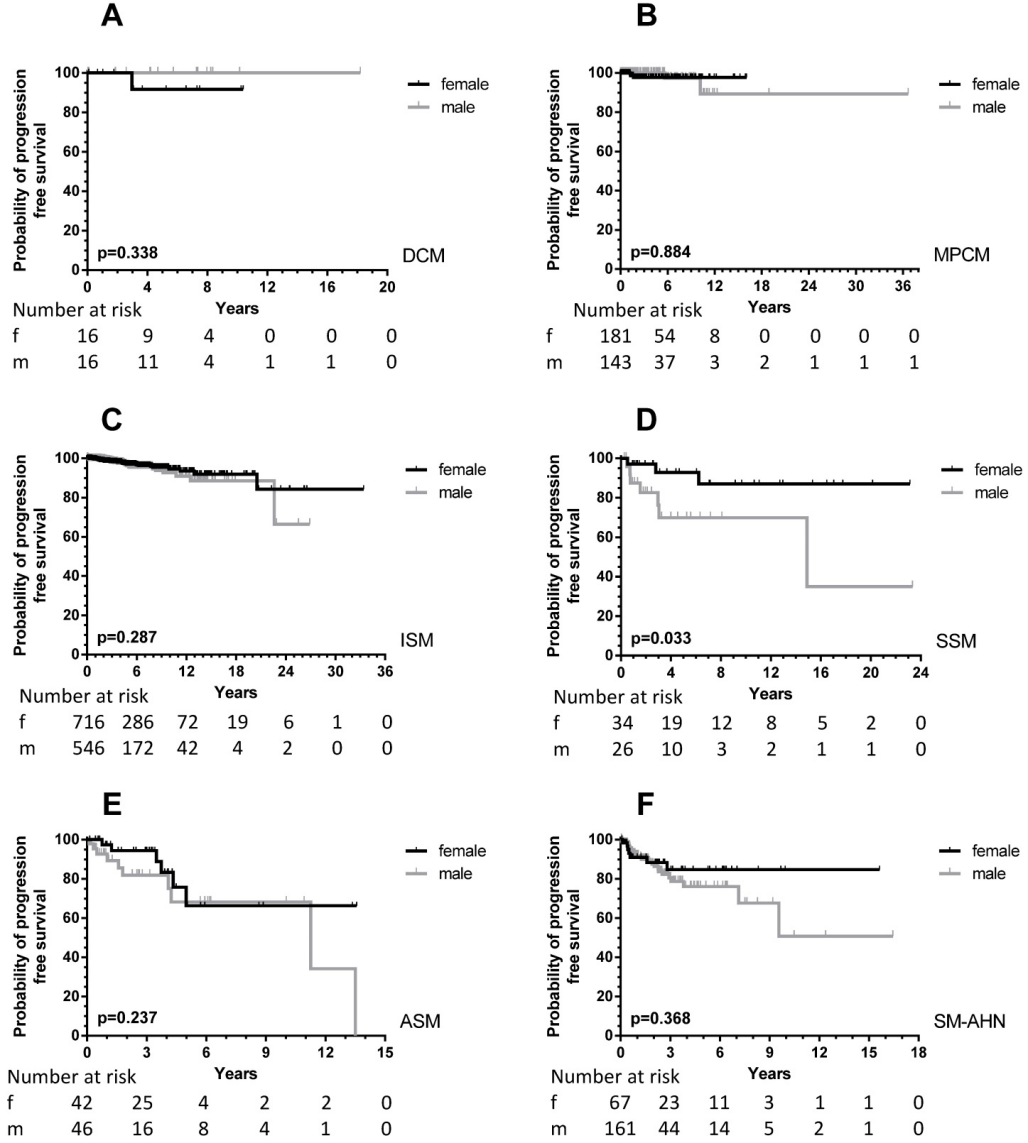
PFS according to sex differences in the various WHO subcategories of all patients (adult and children) with SM. Patients with MIS and MCL were excluded, see Methods. For the definition of progression: see Methods. Grey lines: male patients; black lines: female patients. The numbers at risk are given at the time points outlined on the X-axis. **A:** DCM: diffuse cutaneous mastocytosis; **B:** MPCM: maculopapular cutaneous mastocytosis; **C:** ISM: indolent systemic mastocytosis; **D:** SSM: smoldering systemic mastocytosis; **E:** ASM: aggressive systemic mastocytosis; **F:** SM-AHN: systemic mastocytosis with an associated hematological neoplasm.

With the exception of MIS and MCL, all patients were included in PFS analyses. In total, 64 male (6.0%) and 43 female (3.7%) patients progressed into a more unfavorable WHO subcategory or AHN subgroup. This male predominance was observed in ISM (male, 21/756, 3.7% *vs.* female, 23/974, 2.9%), but also in SM-AHN (male, 24/197, 12.2% *vs.* female, 7/86, 8.1%). In the smaller WHO subgroups, these percentages were 23.3% *vs*. 7.7% for male *vs.* female SSM patients, and 17.2% *vs*. 11.1% for male vs. female ASM patients. In CM, the number of events was rare (one girl with DCM; two girls, one woman and two men with MPCM) and therefore too small to use for comparisons (Figure 3).

In total, a significant difference (*p =* 0.0002) was seen between PFS of male and female patients (Figure 1, right upper part). When children were excluded from this analysis, the significance persisted (*p <* 0.0001). For the children, no difference was seen with the very low number of events. Within most WHO subcategories, significance was lost due to the small numbers of progressing patients except for patients with SSM (*p =* 0.033). However, the curves strongly suggest an earlier progression for male SM-AHN patients (Figure 3).

### Inferior OS in males can only partly be explained by a higher prevalence of SM-AHN

As mentioned above, male patients presented more frequently with AdvSM, which in part explains the worse outcome in males. Of note, even within the SM-AHN group, the OS was worse for male patients (*p =* 0.013, Figure 2). Therefore, we studied the frequency of AHN subcategories at first diagnosis. There was no difference in the frequency of high-risk myeloid neoplasms when comparing male and female patients. In males, 16/197 (8.1%) patients had (secondary) AML and 9/197 (4.6%) patients a high-risk MDS with >5% blasts. Comparable numbers in female patients were 8/86 AML (9.3%) and 2/86 MDS (2.3%). Apart from more female patients with essential thrombocythemia/polycythemia vera (11% *vs.* 3%), most other associated myeloid neoplasms were equally distributed between male and female patients.

Next, we analyzed causes of death within the SM-AHN subcategory, but found no differences either. In male patients, 109/197 died; 73% of deaths were disease-related, 5% therapy-related, 13% other causes, and 9% unknown. In female patients, 32/86 patients died; 72% of deaths were disease related, 6% therapy-related, 19% other causes, and 3% unknown.

Therefore, we asked whether the OS difference between males and females can be explained by an earlier progression and/or unfavorable cytogenetic and molecular profiles.

### Cytogenetic and molecular abnormalities are more frequent in the male cohort

The *KIT* D816V mutation was analyzed in blood and/or BM in 63% (male patients 64%; female patients 61%) of all patients (children included). The mutation was detected in 84% of male and in 75% of the female patients tested (*p <* 0.001).

Cytogenetic analyses were performed in 668 patients (20% of the whole cohort; 64% of these analyses came from patients with cutaneous mastocytosis or indolent forms of SM). All abnormalities were restricted to patients with AdvSM except for 9 patients with ISM and one with SSM. An abnormal karyotype was more frequently identified in males than in females (36/348, 10% *vs.* 16/320, 5%, *p =* 0.006, Table 3).

Detailed additional molecular analyses using NGS were performed in a subset of 190 patients with AdvSM provided by the Mannheim group (Table 4). In this cohort, additional molecular aberrations (apart from *KIT* D816V) were found in 88% of the male patients and in 72% of the female patients (*p =* 0.004). More importantly, high risk molecular mutations (at least one gene mutation in *SRSF2*, *ASXL1*, and/or *RUNX1*, *S/A/R* panel) were more frequently identified in male patients compared to female patients (63% *vs.* 40%; *p =* 0.003; Table 4). The difference was even more impressive if two or more *S/A/R* mutations were taken into account: 28% *vs.* 13% (*p* = 0.027).

## Discussion

A number of disease- and patient-related prognostic variables and several multi-parametric scoring systems have been developed in SM 9-11;18-21. As a result, prognostication improved substantially in the past few years. However, it is still difficult to predict the clinical course and risk of progression in individual patients. More recently, several patient-related factors, including age, have been identified as emerging risk factors and have been included in risk calculations 9,11. We screened for additional patient-related factors in our dataset of the ECNM registry and identified sex as a novel strong and independent prognostic factor in SM. In particular, male patients were found to have an inferior outcome compared to females and were more prevalent in the SM-AHN category. Moreover, we observed that male patients more frequently carried high-risk cytogenetic and molecular aberrations compared to females.

So far, only little is known about the impact of sex on prognosis in various categories of SM. This may be due to the fact that in most previous studies, only a limited number of patients were examined. In the largest cohort of patients with mastocytosis ever collected, the ECNM registry, we could now show that male patients have an inferior outcome concerning OS and PFS. This is best explained by the fact that patients with AdvSM, *e.g.*, aggressive SM, SM-AHN or MCL, were more often male. However, even within such subgroups of AdvSM, namely in ASM and SM-AHN, males had still a worse outcome compared to female patients. Also within the subcategory ISM, OS was worse for male patients, especially during longer follow-up, compared to females.

Obviously, the life expectancy of males in the general population is shorter than of females. This together with the slightly higher age in the male population in our study could in part explain these differences. However, there was also a higher rate of progression in the male patients compared to the female ones, which may indicate more aggressive disease resulting in earlier death.

Skin involvement was more frequent in female mastocytosis patients, whereas organomegaly was more often seen in male patients, which may also help explain the higher percentage of male patients in the AdvSM group. In fact, it is well known, that advanced SM is often associated with organomegaly and with a lack of skin lesions 4,6,22-24.

As far as other disease-related features are concerned, gastrointestinal symptoms and headache were more often reported by women with SM, but all other symptoms, including osteoporosis, did not show a sex preference. The latter is interesting, as osteoporosis per se is a typical female phenomenon, suggesting that a relative increase of osteoporosis in male patients with mastocytosis may account for this sex skewing in this disease population. In a Dutch study examining 157 patients, the prevalence of osteoporosis and/or osteoporotic fractures was even higher in male patients than in female patients, especially in younger individuals (46% *vs.* 18% in patients <50 years) 25.

Only few reports on the clinical impact of demographic features of patients with mastocytosis have been published to date. An epidemiological study from Denmark consisting of 548 patients reported a male prevalence of 40% 26. Within the 7% of patients with AdvSM, no difference in prevalence between males and females was seen, but the numbers of patients were just too small to draw a definitive conclusion. A clear advantage of our ECNM registry-based cohort is that the numbers of patients are much higher in each category of disease, and that in all the contributing centers, experienced hematopathologists contributed to the final diagnosis, which confirmed that no cases with AdvSM were overlooked. The selected series from the Mayo Clinic with 342 patients of whom half belonged to the AdvSM category, showed that in the ISM group, 43% of the patients were male, whereas in the SM-AHN group 70% were male 11. These percentages are remarkably similar to the data obtained in our much larger cohort. A systematic review on 1747 children showed a male predominance with a male-female ratio of 1.4 27. When we analyzed the 397 children in the ECNM registry, we found an almost similar male-female ratio of 1.35. In the subgroup of children with a mastocytoma, boys dominated even more with a ratio of 1.8.

To explain why male patients more often develop AdvSM and even within this category have a poor outcome compared to female patients, we analyzed causes of death and the distribution of the AHN subcategories, but found no difference between male and female patients with almost identical percentages of high risk AHN. However, an abnormal karyotype, considered to be a high-risk feature 14,28, was more frequently identified in males than in females. In a Mayo clinic series of 348 patients, 53 patients with an abnormal karyotype were detected. Clinical correlative studies disclosed significant associations between abnormal karyotype and male sex 29. We found that high-risk molecular mutations (S/A/R gene panel) occurred about twice as often in males than in female patients, which might explain the worse outcome as these mutations are highly predictive for OS 10. It would have strengthened our result if the molecular data in relation to sex differences had been confirmed in other cohorts of advSM patients. Unfortunately, even in the cohort of patients from the Mayo clinics, in which a NGS-based prognostic model for survival was proposed, sex was not included as separate item in their scoring system 30,31.

One can only speculate why male patients with SM more often develop mutations that are associated with high-risk hematological neoplasms. One possibility would be that certain sex hormones facilitate the occurrence of more mutations - for example by introducing clonal instability or by suppressing certain cells or molecules relevant to immune surveillance. An alternative explanation may be that life style factors, such as smoking habits, have an impact on the acquisition of (more) mutations in neoplastic progenitor cells in SM. Indeed, smoking habits and male sex have recently been associated with the occurrence of myeloid mutations in cases with clonal hematopoiesis of indeterminate potential (CHIP) 32. Jaiswal *et al* demonstrated that healthy men above 60 years had a significantly higher likelihood to develop a detectable mutation than women with an odds ratio of 1.3 (95% CI 1.1 to 1.5; *p =* 0.0005) 33. Interestingly, splicing genes (*e.g. SRSF2*) and *ASXL1* were among the most affected genes in humans with clonal hematopoiesis 34. Whether these mechanisms are also causatively involved in the higher prevalence of male patients with AdvSM and high risk mutations (some of which are CHIP-type mutations as well) remains at present unknown. Unfortunately, data on smoking habits have not been collected in the ECNM registry.

A weak point of our study is the retrospective nature of data collection in our registry. Especially the date of first symptoms and date of first visit to the local hospital were retrospectively collected, which made it difficult to define the exact date of first diagnosis. Although most patients could at least exactly define when they first noticed their skin lesions, defining the real date of onset of disease in patients with ISM without skin lesions is a challenge.

Another point of criticism could be that male patients might delay their first visit once symptoms, especially skin symptoms, occur. However, we could not find any indication that male patients postponed their first visits, because the interval between date of first symptoms and date of first visit was median 2356 days for female patients and 1639 days for male patients. Furthermore, overdiagnosis of ISM patients with specific symptoms could have occurred. For example, bias could occur if patients with anaphylaxis or skin lesions more frequently presented in allergy and dermatology centers, respectively. Similarly, ISM patients with severe osteoporosis could have been more often recognized in centers specialized in bone diseases. However, we are confident that the different medical specialties of European centers that contributed to the registry, *e.g.*, hematology, internal medicine, dermatology, gastroenterology, allergy, and pediatrics will have prevented too much selection.

In some adult patients with mastocytosis, the tests required for a full diagnosis and WHO classification - especially bone marrow analysis including flow cytometry and *KIT* mutation analysis - were not always performed. This might explain the rather large number of adult patients with the pre-diagnostic subclassification MIS. On purpose, we omitted these patients from PFS analysis. Additional complex tests, such as mutation analysis were mostly performed in patients with AdvSM, and in centers enabling these tests. In those specialized hematology centers, however, missing data were few and could well be used for our analysis.

Finally, we considered it unlikely that with the new therapeutic modalities, such as midostaurin, PFS and OS would change in relation to sex differences. In this cohort that closed in early 2019, the number of patients treated by midostaurin was still low. However, from the world-wide study of midostaurin in AdvSM published in 2016, there was no sex preference in relation to outcome 35.

## Conclusions

Sex differences have a major impact on clinical features, disease progression and survival in patients with mastocytosis, with male patients having an inferior survival compared to females. This in not only explained by the fact that male patients more often present with SM-AHN, but also that these patients more frequently develop high-risk cytogenetic and molecular abnormalities.

## Figures and Tables

**Table 1:** Demographics and description of all 3403 patients according to the WHO categories

	Mastocytoma	DCM	MPCM	MIS	ISM	SSM	ASM	SM-AHN	MCL
Number (%) of patients at diagnosis	91 (2.7%)	49 (1.4%)	502 (14.8%)	524 (15.4%)	1730 (50.8%)	69 (2.0%)	112 (3.3%)	283 (8.3%)	43 (1.3%)
% Male	59	51	44	33	44	44	52	70	58
**Age at diagnosis in years; median (range)**									
Male	1 (0-5)	2 (0-59)	3 (0-82)	44 (19-85)	48 (0-82)	61 (30-78)	63 (16-82)	66 (1-87)	60 (35-91)
Female	2 (0-15)	8 (0-68)	26 (0-78)	40 (18-87)	47 (15-83)	52 (25-79)	57 (29-83)	66 (20-87)	51 (27-72)
Tryptase, median (range)	5.9 (1.3-26.4)	15.1 (1.7-103)	7.1 (1-126)	13.8 (1-200)	30 (1-885)	200 (21-2100)	165 (8.9-1432)	135 (1.8-1060)	383 (74.9-4530)
Number of patients with follow-up data	49	32	324	277	1262	61	88	228	36

Abbreviations: DCM: diffuse cutaneous mastocytosis; MPCM: maculopapular cutaneous mastocytosis; MIS: mastocytosis in the skin; ISM: indolent systemic mastocytosis; SSM: smoldering systemic mastocytosis; ASM: aggressive systemic mastocytosis; SM-AHN: systemic mastocytosis with an associated hematologic neoplasm; MCL, mast cell leukemia; WHO, World Health Organization.

**Table 2:** Signs and symptoms in relation to sex difference in patients with mastocytosis

Organ involvement or symptom^@^	yes	no	*P*^$ univariate^	*P*^^multivariate^
**Skin involvement**				
Male n (%)	1087 (71^&^)	452 (29)	0.0001	0.0001
Female n (%)	1604 (86)	262 (14)		
**Enlarged spleen**				
Male n (%)	249 (18)	1161 (82)	<0.0001	not tested
Female n (%)	138 (8)	1585 (92)		
**Enlarged liver**				
Male n (%)	224 (16)	1181 (84)	<0.0001	not tested
Female n (%)	140 (8)	1581 (92)		
**Enlarged lymph nodes**				
Male n (%)	125 (6)	1189 (90)	<0.0001	not tested
Female n (%)	86 (5)	1537 (95)		
**Organomegaly^#^**				
Male n (%)	335 (23)	1092 (77)	<0.0001	<0.0001
Female n (%)	217 (13)	1514 (87)		
**Skin symptoms including flushes**				
Male n (%)	851 (58)	612 (42)	<0.0001	<0.0001
Female n (%)	1370 (76)	436 (24)		
**Anaphylaxis**				
Male n (%)	309 (22)	1101 (78)	0.240	0.232
Female n (%)	403 (24)	1292 (76)		
**Bone signs and symptoms (pain, osteoporosis, osteopenia)**				
Male n (%)	430 (34)	820 (66)	0.232	0.788
Female n (%)	563 (37)	969 (63)		
**Gastrointestinal symptoms**				
Male n (%)	446 (31)	983 (69)	<0.0001	<0.0001
Female n (%)	767 (44)	987 (56)		
**Headache**				
Male n (%)	121 (8)	1418 (92)	<0.0001	0.001
Female n (%)	291 (16)	1575 (84)		
**Allergy**				
Male n (%)	418 (34)	829 (66)	0.436	0.662
Female n (%)	475 (32)	1004 (68)		

@ Signs and symptoms were not documented in all patients # Any of the three: enlarged spleen, liver or lymph nodes & Percentages were rounded-up to enhance clarity $ P-value assessed by univariate (Fischer exact test) and ^multinominal logistic regression including the WHO subcategory.

**Table 3:** Cytogenetic abnormalities in all patients with SM

WHO sub-classification	Male = 348 (total number tested)	Female n = 320 (total number tested)
ISM	46, XY, t(2;22) (p23; q11.2~12) 8/46, XY 12	46, XX, inv(2)
	45, XY, rob (13;14)	46, XX, t(5;6) (q?23, q?16)
	45, X, -Y	46, XX, del(12) (p13)
	47, XY, +Y	46, XX, del(20) (q?) 3/46, XX 16
	47, XY, +Y	
SSM	45, X, -Y	
ASM	46, XY, del(20) (q11.2q13) 18/46, XY 9	46, XX, iso17(q10)
	47, XY, +X	46, XX, t(5;12) (q33; p13)
SM-AHN	47, XY, der(1;19) (q10; p10)	45, XX-7 5/46, XX 20
	40, XY, der(1)(q), der(2)(p), der(3)(p), -3,-4,der(7)(q), der(7)(p), -8,-9,-10,-11,der(11)(p), -13,-18,+mar complex karyotype	46, XX, t(8; 21) (with AML as AHN)
	46, XY, t(2;2) (p23; q32) 10/47, XY, t(2;2) (p23, q32), +8 2/46, XY 9	46, XX, t(8; 21) (with AML as AHN)
	46, XY, t(2;15) (p16; q15)	47, XX, +8
	45, XY, -7	46, XX, t(9; 14)
	45, XY, -7 3/46 XY 1	46, XX, del (13q14)
	45, XY, -7 10/46, XY 10	
	45, XY, inv(3), -7	
	45, XY, -7 16/48, XY, +8, +19 4/46, XY 2	
	46, XY, t(7; 9; 11) (q35; p11; q23), del(7) (q32)	
	47, XY, +8	
	47, XY, +8	
	47, XY, +8, del(12) (p12p13)	
	46, XY, inv(11)	
	46, XY, del(12) (p11p13)	
	46, XY, t(12; 22)	
	47, XY, +19 12, 46, XY 8	
	47, XY, del(20) (q11.22q13), +22	
	46, XY, del(20) (q12) 15/46, XY 3	
	45, X, -Y	
	45, X, -Y	
	45, X, -Y	
	45, X, -Y 2; 46, XY 10	
	45, X, -Y	
	45, X, -Y 16/46, XY 9	
	47, XXY	
	47, XXY	
MCL	45, X, -Y 16/46, XY 4	46, XX, del(8)(q21), der(10) t(8; 10)(q22; q26), r(12) (p12q15), der(16) t(12;16) (q21; q22), inv(12) (q21q24) 8/46, XX 16
	40, XY der(1)(q), der(2)(p), der(3)(p), -3,-4,der(7)(q), der(7)(p), -8,-9,-10,-11,der(11)(p), -13, -18, +mar complex karyotype	46, XX(del12) (p13p13)
		complex aberrant
		complex aberrant

Abbreviations: ISM, indolent systemic mastocytosis; SSM, smoldering systemic mastocytosis; ASM, aggressive systemic mastocytosis; SM-AHN, SM with associated hematologic neoplasm; MCL, mast cell leukemia.

**Table 4:** Detailed molecular analysis of the Mannheim cohort of 190 patients with advanced SM

Characteristics	Male (n = 127)	Female (n = 63)	*P*
Age, years; median (range)	69 (25-90)	64 (24-83)	0.009
**WHO diagnosis, n (%)**			
ASM	6 (5)	10 (16)	0.013
SM-AHN	106 (83)	48 (76)	0.243
MCL (± AHN)	15 (12)	5 (8)	0.464
Leukemic transformation^a^, n (%)	21 (17)	12 (19)	0.687
**Mast cell infiltration in BM, histology (%)**			
Median (range)	30 (5-95)	25 (5-100)	0.616
**Serum tryptase, µg/L**			
Median (range)	170 (4-1854)	180 (5-1690)	0.835
**Driver mutation, n (%)**			
*KIT* D816V	119 (94)	56 (89)	0.263
Other *KIT* mutations	2 (2)^b^	4 (6)^c^	0.095
No *KIT* mutations	6 (5)	3 (5)	1.0
**Any additional somatic mutations,**			
**n (%)** ≥ 1 additional mutation(s)	112 (88)	44 (70)	0.004
**S/A/R^d^ mutation(s), n (%)**			
≥ 1 S/A/R mutation(s)	80 (63)	25 (40)	0.003
≥ 2 S/A/R mutations	35 (28)	8 (13)	0.027
Aberrant karyotype^e^, n (%)	18 (17)	9 (19)	1.0
**Follow-up, years**			
Median (range)	2.1 (0-17)	2.6 (0-21)	0.083
Death, n (%)	69 (54)	26 (41)	-
**Overall survival, years**			
Median (95% CI)	2.9 (1.9-3.9)	4.6 (2.7-6.5)	0.027

Abbreviations: AHN, associated hematologic neoplasm; ASM, aggressive systemic mastocytosis; BM, bone marrow; MCL, mast cell leukemia; WHO, World Health Organization. ^a^ to secondary MCL (±AHN) or secondary SM-AML; ^b^* KIT* D816H, n = 1; *KIT* F522C, n = 1; ^c^ *KIT* D816H, n = 2; *KIT* D816Y, n = 2; ^d^ gene mutation(s) in *SRSF2*, *ASXL1* and/or *RUNX1* (S/A/R) panel; ^e^ data available in n = 168.

## Abbreviations

AdvSM: advanced systemic mastocytosis
AML: acute myeloid leukemia
ASM: aggressive systemic mastocytosis
BM: bone marrow
CHIP: clonal hematopoiesis of indeterminate potential
CI: confidence interval
CM: cutaneous mastocytosis
DCM: diffuse cutaneous mastocytosis
ECNM: European competence network on mastocytosis
GI: gastrointestinal tract
ISM: indolent systemic mastocytosis
MCL: mast cell leukemia
MDS: myelodysplastic syndrome
MIS: mastocytosis in the skin
MPCM: maculopapular cutaneous mastocytosis
OR: odds ratio
OS: overall survival
PFS: progression-free survival
S/A/R panel: *SRSF2/ASXL1/RUNX1* panel
SM: systemic mastocytosis
SM-AHN: systemic mastocytosis with an associated hematologic neoplasm
SSM: smoldering mastocytosis
WHO: World Health Organization
